# Natural History of Cone Disease in the Murine Model of Leber Congenital Amaurosis Due to *CEP290* Mutation: Determining the Timing and Expectation of Therapy

**DOI:** 10.1371/journal.pone.0092928

**Published:** 2014-03-26

**Authors:** Shannon E. Boye, Wei-Chieh Huang, Alejandro J. Roman, Alexander Sumaroka, Sanford L. Boye, Renee C. Ryals, Melani B. Olivares, Qing Ruan, Budd A. Tucker, Edwin M. Stone, Anand Swaroop, Artur V. Cideciyan, William W. Hauswirth, Samuel G. Jacobson

**Affiliations:** 1 Department of Ophthalmology, College of Medicine, University of Florida, Gainesville, Florida, United States of America; 2 Scheie Eye Institute, Department of Ophthalmology, University of Pennsylvania, Philadelphia, Pennsylvania, United States of America; 3 Stephen A. Wynn Institute for Vision Research, University of Iowa Carver College of Medicine, Iowa City, Iowa, United States of America; 4 Howard Hughes Medical Institute, University of Iowa Carver College of Medicine, Iowa City, Iowa, United States of America; 5 Neurobiology-Neurodegeneration & Repair Laboratory, National Eye Institute, National Institutes of Health, Bethesda, Maryland, United States of America; University Zürich, Switzerland

## Abstract

**Background:**

Mutations in the *CEP290* (cilia-centrosomal protein 290 kDa) gene in Leber congenital amaurosis (LCA) cause early onset visual loss but retained cone photoreceptors in the fovea, which is the potential therapeutic target. A cone-only mouse model carrying a C*ep290* gene mutation, *rd16;Nrl^−/−^*, was engineered to mimic the human disease. In the current study, we determined the natural history of retinal structure and function in this murine model to permit design of pre-clinical proof-of-concept studies and allow progress to be made toward human therapy. Analyses of retinal structure and visual function in *CEP290-*LCA patients were also performed for comparison with the results in the model.

**Methods:**

*Rd16;Nrl^−/−^* mice were studied in the first 90 days of life with optical coherence tomography (OCT), electroretinography (ERG), retinal histopathology and immunocytochemistry. Structure and function data from a cohort of patients with *CEP290*-LCA (n = 15; ages 7–48) were compared with those of the model.

**Results:**

*CEP290*-LCA patients retain a central island of photoreceptors with normal thickness at the fovea (despite severe visual loss); the extent of this island declined slowly with age. The *rd16;Nrl^−/−^* model also showed a relatively slow photoreceptor layer decline in thickness with ∼80% remaining at 3 months. The number of pseudorosettes also became reduced. By comparison to single mutant *Nrl^−/−^* mice, UV- and M-cone ERGs of *rd16;Nrl^−/−^* were at least 1 log unit reduced at 1 month of age and declined further over the 3 months of monitoring. Expression of GNAT2 and S-opsin also decreased with age.

**Conclusions:**

The natural history of early loss of photoreceptor function with retained cone cell nuclei is common to both *CEP290*-LCA patients and the *rd16;Nrl^−/−^* murine model. Pre-clinical proof-of-concept studies for uniocular therapies would seem most appropriate to begin with intervention at P35–40 and re-study after one month by assaying interocular difference in the UV-cone ERG.

## Introduction

Leber congenital amaurosis (LCA) is a molecularly heterogeneous group of severe early-onset human diseases [1], [2]. By clinical definition, LCA patients show severe visual loss early in life, but the morphological basis of the malfunction differs among genotypes [3]. Visual loss can be due to severe outer retinal degeneration or retinal disorganization with loss of photoreceptors in early life. A few molecular forms of LCA, however, have unexpectedly shown retained outer retinal structure, indicating structure-function dissociation. For example, the form of LCA associated with *RPE65* (retinal pigment epithelium-specific-65-kDa) gene mutations can have retained photoreceptors [4] and treating regions with residual retinal pigment epithelium (and photoreceptors) with viral-mediated gene augmentation has led to remarkable increases in vision. The road to clinical trials of *RPE65*-LCA was paved by critical proof-of-concept research with large and small animal models of the human disease [5].

In another molecular form of LCA with severe visual consequences, *CEP290* (cilia-centrosomal protein 290 kDa)-LCA, patients over a wide age range were shown to retain a central island of dysfunctional cone photoreceptors [3], [6]–[8]. Early loss of rod photoreceptors in *CEP290*-LCA has been documented [6], [7]. The survival of the central cones into adulthood makes *CEP290*-LCA a worthy target for a macular gene augmentation therapy. Murine models of the disease recapitulate the rapid rod cell loss of the human disease [9], [10] but the high rod:cone ratio in the mouse retina complicates proof-of-concept research of cone-selective therapy. For that reason, a murine model was genetically-engineered to more closely approximate the human disease. An all-cone retina with *Cep290* mutation, the double mutant *rd16;Nrl^−/−^*, showed dissociation of cone structure and function (as in the human disease); this determination was made in 3–4-months-old mice only [7].

Many questions still need to be answered to enable proof-of concept studies for therapy to be performed in this model of human *CEP290*-LCA? What is the natural history of the cone disease in this murine model? What would be the optimal timing of therapeutic intervention in the model? What are the expectations from such treatment and how long should we wait for a postulated effect? The present work studies the natural history of cone structure and function in the *rd16;Nrl^−/−^* mouse, confronts the complexity of the photoreceptor dysmorphogenesis well known to *Nrl^−/−^* mice; determines the window of timing for testing therapies; and establishes outcomes for determining efficacy. The results should expedite the pre-clinical steps toward eventual translation to clinical trials.

## Materials and Methods

### Human Subjects and Ethics Statement

The human studies were approved by the University of Pennsylvania Institutional Review Board (IRB; Protocol 226100). For adult subjects, written informed consent was obtained. For all children, written parental permission was obtained. Written assent was obtained from children ages 12 to 17; oral assent was obtained from children ages 7 to 11; children under the age of 7 years were enrolled with written parental permission. These consent/assent procedures were approved by the Institutional review board and the procedures adhered to the tenets of the Declaration of Helsinki. The study included 15 patients with LCA due to *CEP290* mutations (some phenotypic features of 14 of the patients have been published) [7]; the additional patient was a 9-year-old girl who is a compound heterozygote for IVS26+1655 A>G and IVS13+1 G>C mutations. There were also 26 patients with retinal degeneration including simplex/multiplex retinitis pigmentosa (RP), X-linked RP and Usher syndrome. Patients underwent a complete eye examination, including visual acuity and optical coherence tomography (OCT). Data will be made freely available upon request.

Retinal cross-sectional imaging with 9-mm (30-degree) line scans along the horizontal meridian crossing the fovea was mainly performed with a spectral-domain OCT unit (RTVue-100; Optovue, Fremont, CA); images from a subset of patients were acquired using a time-domain OCT unit (OCT3; Carl Zeiss Meditec, Dublin, CA). Two novel parameters of foveal structure were derived from *CEP290*-LCA OCT data that were mostly (14 of 15) collected during previous studies [3], [6], [7]. The first parameter was average ONL thickness of the foveal region which was defined as the average of 5 samples obtained at the foveal pit and two on each side of the fovea along the vertical meridian at 0.15 and 0.3 mm eccentricity. The second parameter was the width of the foveal ONL island which was defined as the retinal distance between two points at half maximum ONL.

### Animals and Ethics Statement

All experiments were performed in strict accordance with the recommendations in the Guide for Care and Use of Laboratory Animals of the National Institutes of Health and the USDA’s Animal Welfare Act and Animal Welfare Regulations, and complied with the ARVO Statement for the Use of Animals in Ophthalmic and Vision Research. The protocols were approved by the Institutional Animal Care and Use Committee of the University of Florida (IACUC Protocol # 201304739) and the University of Pennsylvania (IACUC Protocol #804387). Data will be made freely available upon request. The *rd16;Nrl^−/−^* double mutant mice and C57BL/6 control mice (Jackson Laboratory, Bar Harbor, ME) were maintained in the University of Florida Health Science Center's animal care facilities in controlled ambient illumination on a 12-hour light/12-hour dark cycle (ambient illumination, <3 lux). Access to food and water was ad libitum.

### Electroretinography

ERGs were performed with published methods [7], [11], [12] using general anesthesia (ketamine HCl, 65 mg/kg, xylazine, 5 mg/kg, intraperitoneal injection) and topical corneal anesthetic (proparacaine HCl). Pupils were dilated (tropicamide 1%, phenylephrine 2.5%). A computer-based system (Espion, Diagnosys LLC, Littleton, MA, USA) was used to record light-adapted (40 cd.m^−2^ white) ERGs in response to a Xenon UV flash (360 nm peak, Hoya U-360 filter, Edmund Optics, Barrington, NJ, USA) and to a green flash produced with LEDs (510 nm peak; 0.87 log phot-cd.s.m^−2^, 4 ms). The energy of the UV flashes was adjusted to evoke responses matched in waveform to those elicited by the green stimulus in WT mice. The stimuli were presented in a ganzfeld lined with aluminum foil [7], [11], [12].

### Optical Coherence Tomography

Retinal OCT imaging was performed as previously described [12], [13]. Retinal cross-sectional images of *rd16;Nrl^−/−^* and control mice were acquired with a spectral domain (SD) OCT system (Bioptigen, Inc., Durham, NC). Animals were anesthetized and pupils were dilated as for ERG recordings. Corneas were lubricated during the imaging session (Systane Ultra ophthalmic lubricant; Alcon Ltd., Fort Worth, TX). The optic nerve head (ONH) was centered within a 1.5 mm diameter field of view under fast fundus mode, using a raster consisting of 200 parallel b-scans of 200 longitudinal reflectivity profiles (LRPs) per scan line [13]. High-resolution scans (40 parallel b-scans of 1000 LRPs in each scan line, each repeated four times) were acquired along the horizontal (nasal-temporal) and vertical (dorsal-ventral; superior-inferior) axes. Repositioning of the eyes then occurred and the ONH was placed at top or bottom center of the view. High-resolution scans were repeated at these locations for coverage up to 3.2 mm.

Post-acquisition processing of OCT data was performed with custom programs (MATLAB 7.5; MathWorks, Natick, MA) and commercial software (InVivoVue Clinic software; Bioptigen, Inc.). Four repetitions of the high-resolution scans were averaged using the manufacturer’s software. Vertical scans with ONH at the center, and those superior and inferior to the center of the ONH were montaged by custom programs. LRPs of the merged OCT images were aligned by manually straightening the retinal pigment epithelium (RPE) reflection [12], which was defined as the second hyperreflective band from the sclerad side [14]. Retinal thickness is the distance from the vitreoretinal interface and the RPE peak. Photoreceptor (termed ONL+) thickness is defined as the distance between the signal trough delimited by the signal peak defining the sclerad side of the outer plexiform layer to the signal trough vitreal to the hyperreflective band of the RPE. When data are represented as profiles across the vertical meridian, the average and SD values are calculated from all data points in the vertical profile excluding 0.18 mm around ONH. Photoreceptor ONL+ and ONL fractions, OCT and histology measurements respectively, were estimated by normalizing to the mean values of the youngest age studied (P31 for OCT, n = 10 eyes, and P21–40 for histology, n = 6 eyes, measurements).

Retinal *en face* reflectance images to enable counts of pseudorosettes were generated using 350 by 350 horizontal raster scans centered within the 1.5 mm diameter field of view. Spatial distribution of pseudorosettes around the ONH was revealed by integrating the OCT backscatter intensity between two boundaries that envelope all rosettes in the OCT raster images. The light-scattering pseudorosettes appear as white spots in the *en face* image. Total numbers of pseudorosettes in the sampled area were counted manually using ImageJ (http://rsb.info.nih.gov/ij/). Comparisons between groups were performed using unpaired, two-tailed t-tests. Significance is defined as a *p* value <0.05. Density of pseudorosettes in different sectors within the central retinal region was also quantified (n = 8 eyes for two age groups).

### Histopathology and Immunocytochemistry

Eyes of *rd16;Nrl^−/−^* mice were enucleated at postnatal day 21 (P21), P40, P60 and P80 and immediately placed in 4% paraformaldehyde for 24 hours. P60 WT mice were used as controls. Following incubation in 30% sucrose/1X PBS (2 hrs), tissue was prepared for cryoprotection and sectioning as previously described [15]. Ten micron sections were cut on a cryostat (Leica CM3050 S, Wetzlar, Germany). Sections designated for H&E staining were rehydrated for 10 minutes, followed by immersion in 95% EtOH for 2 minutes, 70% EtOH for 2 minutes and a wash with distilled H_2_O. Harris’s hematoxylin solution (RICCA Cat. No. 3530-1, Arlington, TX) was applied for 6 minutes followed by a 1 minute wash in running tap water. 0.2% ammonia “bluing” solution (Fisher chemicals #A669-500, Waltham, MA 02454) was applied for 45 seconds, followed by a wash in running tap water. This was followed with ten successive immersions in 95% EtOH and a counterstain in eosin Y solution (RICCA Cat. No. 2850-16, Arlington, TX) for 6 minutes. Sections were then dehydrated with 95% EtOH and 2 immersions in absolute EtOH for 5 minutes each. Following immersion in deionized H_2_O, AQUA-MOUNT mounting media was applied and slides were coverslipped. Sections designed for immunohistochemistry were processed as follows. After rinsing with 1X PBS, sections were incubated with 0.5% Triton X−100 for 1 hour followed by a 30 min incubation with a blocking solution of 1% bovine serum albumin (BSA). Retinal sections were then incubated overnight at 4°C with rabbit polyclonal antibodies raised against cone transducin alpha (GNAT2) (sc-390, Santa Cruz, 1∶500) or S cone opsin (AB5407, Chemicon International, 1∶300) and lectin PNA conjugated to Alexa Fluor 594 (L32459, Invitrogen, 1∶400) diluted in 0.3% Triton X−100/1% BSA. The following day, sections were rinsed with 1X PBS and incubated for one hour at room temperature in anti-rabbit IgG secondary antibody Alexa-fluor 488 (Invitrogen, Eugene, Oregon, Cat#A11008) diluted in 1X PBS at 1∶500. After a wash, sections were cover slipped with SlowFade Gold antifade reagent with DAPI (S36939, Life Technologies). Retinal sections were imaged at 5X using a fluorescent Axiophot microscope (Zeiss, Thornwood, NY). 20X images were captured with spinning disk confocal microscopy (Nikon Eclipse TE2000 microscope equipped with Perkin Elmer Ultraview Modular Laser System and Hamamatsu O-RCA-R2 camera). Exposure settings were consistent across images at each magnification.

Pseudorosettes were counted in sections from cuts through the globe representing the far peripheral retina in three *rd16;Nrl^−/−^* mice each from the different age groups; comparison with any regional differences from OCT was not possible because attention to exact dorsal-ventral orientation of the tissue did not occur. ONL thickness was measured using Volocity software (Perkin Elmer) in two peripheral retinal cuts from three *rd16;Nrl^−/−^* animals. Counts were made in four equally-spaced quadrants in each far peripheral section. Peripheral ONL thickness measurements and pseudorosette counts were averaged and compared across ages using unpaired, two-tailed t-tests. Significance is defined as a *p* value <0.05.

## Results

### Structure and Function in Human *CEP290*-LCA

**Figure 1:**
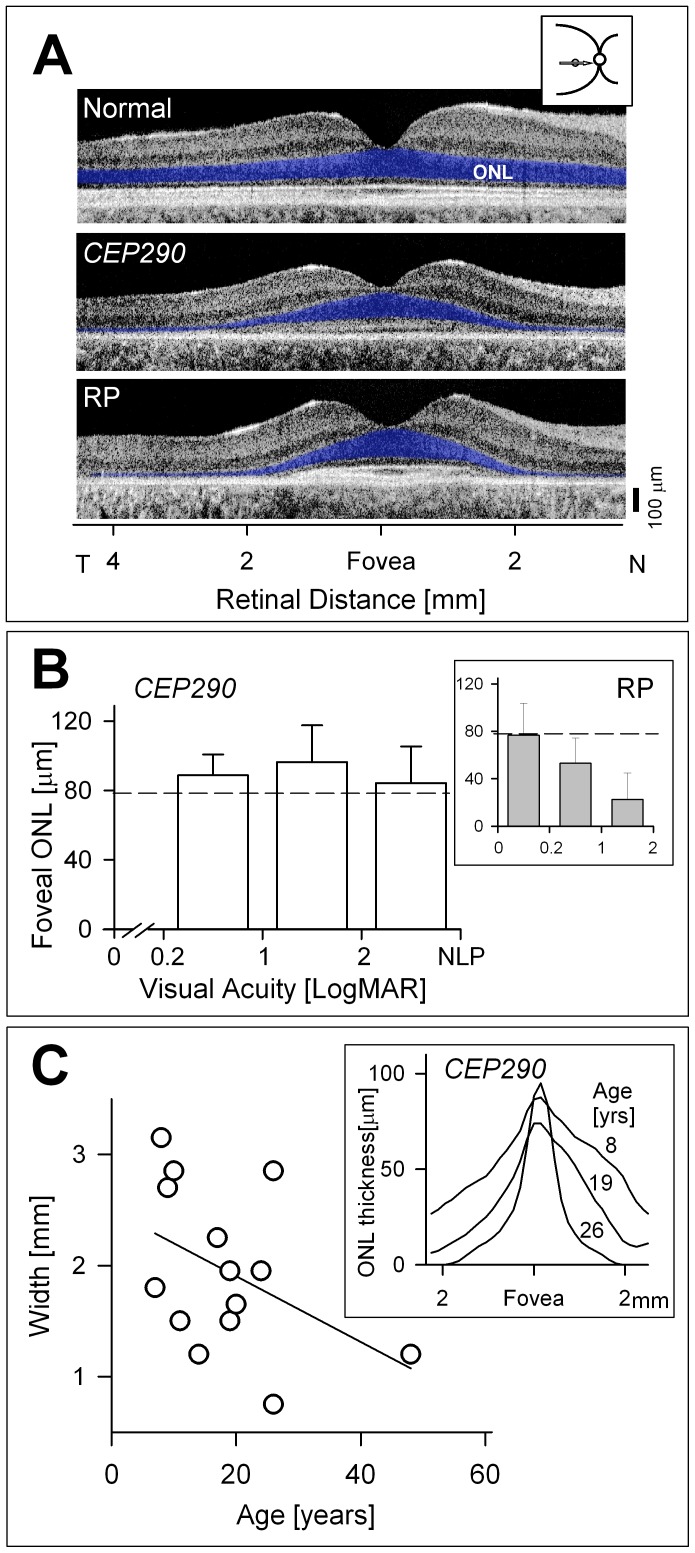
Structure and function in the central retina of *CEP290*-LCA patients. (A) Cross-sectional OCT scans along the horizontal meridian through the fovea in a normal subject, a *CEP290*-LCA patient, and an RP patient. ONL is highlighted in blue. Inset shows location of scan. (B) Relationship of foveal ONL thickness and visual acuity in *CEP290*-LCA patients. Bar graph represents the average ±1SD foveal ONL thickness of eyes in the different visual acuity ranges (n = 3, for 0.1–1 LogMAR; n = 5, for 1–2 LogMAR; and n = 11, for 2–NLP). Dashed line is lower limit of normal and emphasizes that despite low acuities, foveal ONL is within normal limits. Inset, data from a series of RP patients plotted similarly to show the more expected relationship between structure and visual acuity in retinal degenerations (n = 3, for 0–0.2 LogMAR; n = 20, for 0.2–1 LogMAR; and n = 3 for 1–2 LogMAR). Dashed line is also lower limit of normal. (C) Relationship in *CEP290*-LCA patients of width of the ONL in the central retina and patient age at time of examination. ONL width was unable to be defined in a 32-year-old *CEP290*-LCA patient with maculopathy. Solid line is linear regression. Inset, traced central ONL peaks in representative patients of different ages.

LCA caused by *CEP290* mutations can show a wide spectrum of visual acuity results but, in general, most of the measurable values are severely reduced [16], [17]. It was thus surprising when we observed by OCT retinal imaging that there were foveal islands of retained photoreceptor nuclei in nearly all such patients [3], [6], [7]. A cross-sectional image horizontally across the retina through the fovea of a 23-year-old normal subject with logMAR of 0 (Snellen, 20/20) shows a continuous photoreceptor outer nuclear layer (ONL) with adjacent laminar architecture representing synaptic and nuclear layers to the vitreal side, and inner and outer segment and RPE layers to the choroidal side (Fig. 1A, upper panel). In contrast, a 9-year-old LCA patient with no light perception (NLP) and mutant *CEP290* alleles retains a central island of ONL in the cone-rich fovea that is within normal limits for thickness, although the inner and outer segment laminar architecture is abnormal (Fig. 1A, middle panel) [7]. There is no measurable ONL beyond the central island. An RP patient (age 17) shows a similar central island of ONL, but has a visual acuity of 0.3 (20/40; Fig. 1A, lower panel); the distal laminar architecture representing outer and inner segments approximates that of the normal subject in the very central retina.

To enable comparisons to be made between human *CEP290*-LCA function and structure and that of the *rd16;Nrl^−/−^* mouse model, we plotted the relationship between visual acuity and foveal ONL thickness in a cohort of *CEP290*-LCA patients (n = 19 eyes of 15 patients, ages 7–48 years; Fig. 1B) [7]. Eleven of the 19 eyes had a foveal ONL thickness of >79.3 μm, which represents the lower limit of normal (mean ± SD, 93.7±7.2 μm; n = 10). There was a wide range of abnormal visual acuities (from 0.4 logMAR to NLP) but 11/19 eyes (57%) of the 15 patients in this cohort have HM (hand motions) vision or worse. Linear regression through these *CEP290*-LCA patient data had a slope of −0.02 (r^2^, 0.027) and this was not significantly different from 0 (p = 0.49). In other words, there was no decrease in foveal ONL thickness associated with lower acuities. This is in contrast to the data from a group of patients with other retinal degenerative diseases, collectively termed RP (n = 26 patients; ages 10–69; Fig. 1B, inset). Unlike in *CEP290*-LCA, the RP patients show the more expected relationship: reduction in visual acuity is accompanied by decreasing foveal ONL thickness [4], [18].

Does the island of cone photoreceptor nuclei remain stable with age in *CEP290*-LCA? Without longitudinal data available in our cohort, we used cross-sectional data to quantify the width of the central island along the horizontal meridian in 14 patients (Fig. 1C). The width of the central island tends to decrease with age (slope of −1.96; r^2^, 0.19), suggesting that a slow degenerative process is likely ongoing in these patients, as it is in all retinal degenerations.

### Retinal Structure in *rd16;Nrl^−/−^* mice at Different Ages

**Figure 2:**
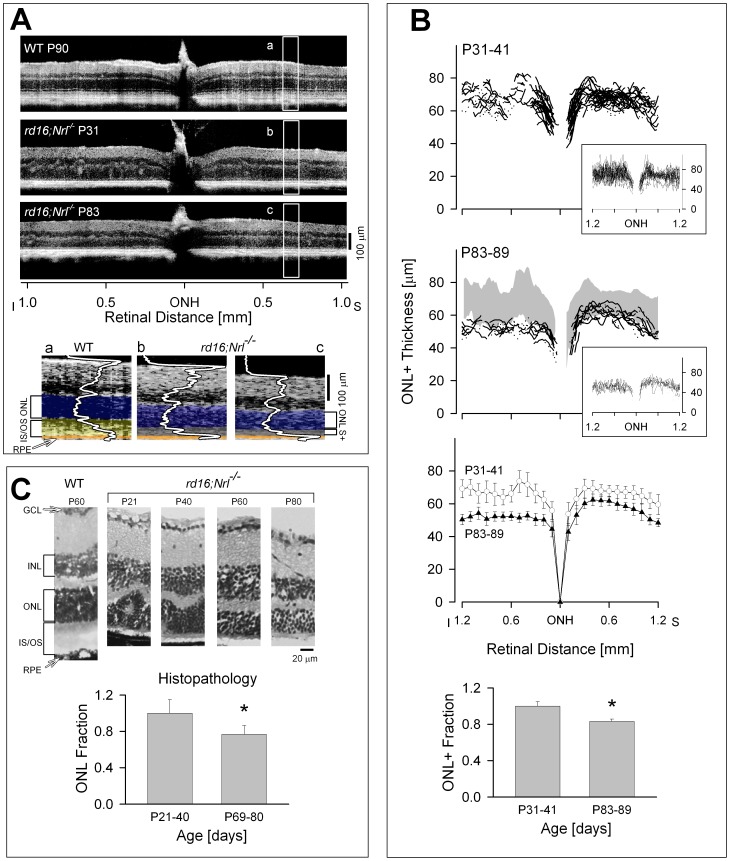
OCT abnormalities in *rd16;Nrl^−/−^* mice. (A) Upper panels: Representative OCT scans vertically across ∼2 mm of retina (centered at the ONH, optic nerve head) in a WT mouse and in two *rd16;Nrl^−/−^* mice of different ages. Lower panels: Magnified parts of the superior region of the retinal sections with overlaid longitudinal reflectivity profiles (LRPs) to demonstrate the reflective abnormalities in the outer retinal region in *rd16;Nrl^−/−^* mice (b and c) compared with C57BL6 WT (a). (B) Upper two panels: Vertical OCT sections quantified for ONL+ thickness in two age groups of *rd16;Nrl^−/−^* mice. Regions of outer retina with pseudorosettes were excluded in the measurement. ONL+ profiles in the older (P83–89, n = 12 eyes) age group were thinner than those in younger (P31–41, n = 35 eyes) mice; gray bands in the P83–89 plot represent mean±2 SD for ONL+ thickness of the P31–41 mice. For reference, insets at lower right of the upper two plots show original raw data before suppression of pseudorosette regions. Third panel from top: Means of ONL+ data across the vertical meridian in two age groups (error bar: ± SD; P31–41, open circles; P83–89, filled triangles). Lowest panel: Histograms showing average ONL+ fraction across vertical meridian of two age groups (*represents *p*<0.001). (C) Histological sections of *rd16;Nrl^−/−^* retina at 4 different ages from P21 to P80, compared with a WT retinal section. Histograms show ONL fraction (based on the earlier age group) in *rd16;Nrl^−/−^* mice from peripheral retina (n = 6 eyes in each of the two age groups, *represents *p* = 0.01).

Optical imaging provided the opportunity to study the retinal morphological changes at different ages in the *rd16;Nrl^−/−^* mice. Representative vertical OCT scans across the ONH are shown from a wild-type (WT) mouse at age 90 days (P90), and *rd16;Nrl^−/−^* mice at P31 and P83 (Fig. 2A). The scans from the mutant mice differ from that of the WT mouse. For example, total retinal thickness differs as well as the thickness of the photoreceptor layers. WT mice, of course, have a different complement of photoreceptors than the *rd16;Nrl^−/−^* mice. WT have mainly rods and a minority of cones, while the mutant mice have exclusively cone photoreceptors [7], [11], [19]. The mutant mice also show hyperreflective loci (presumed pseudorosettes) within the outer retinal layers and extending into the inner retina; the loci are most evident in the inferior retinal part of the section. The hyperreflectivities appear more prominent in P31 than in P83. Magnified sections from the three scans indicate some of the difficulty in defining the outer retinal layers in the *rd16;Nrl^−/−^* mice compared with WT. ONL and the laminae attributed to IS and OS (distal to the OLM) are readily discernible in WT cross-sectional images, as is the RPE hyperreflection [12], [13]. The *rd16;Nrl^−/−^* mice present a different pattern of reflectivities with an indistinct OLM and thus no clear delineation of the laminar architecture of the IS and OS of these cone cells (scattering band distal to ONL is labeled S+) [7]. An RPE hyperreflectivity, however, is discernible (Fig. 2A lower panels). ONL thickness appears reduced in P83 compared to P31.

Photoreceptor layer thickness across the ONH along the vertical meridian (central 2.4 mm of retina) is quantified for two age groups of mutant mice, ages 31–41 and 83–89 days (Fig. 2B). The profiles of photoreceptor laminae at P31–41 appeared spiky because of the disturbances in the outer layers by the pseudorosette structures (Fig. 2A; Fig. 2B, inset near P31–41 panel). The profiles, when areas with pseudorosettes were specifically excluded, however, become smoother as would be expected for a photoreceptor layer [13]. The spiky appearance in the vertical profiles diminishes in the older age group, suggesting a reduction in pseudorosettes with age in the mutants (Fig. 2B, inset near P83–89 panel). The photoreceptor laminae across the vertical meridian in the P31–41 age group is significantly thicker than in the older age group (mean ± SD, P31–41: 66.4±7.6 μm; P83–89: 54.7±7.0 μm; p<0.001), indicating a decline with age in the mutants. When the spiky profile data that included the pseudorosettes was similarly analyzed, there was also a significant difference between ages (P31–41: 69.4±9.7 μm; P83–89: 54.4±7.6 μm; p<0.001). There is an apparent superior-inferior (dorsal-ventral) asymmetry with the P83–89 age group showing greater thinning in the inferior region than superiorly (Fig. 2B). We explored this possible regional variation. First, we compared the inferior and superior photoreceptor layer thicknesses at the P31–41 age group and there was no significant difference (P31–41 Sup: 66.8±4.9 μm; versus Inf: 67±6.3 μm; p = 0.85). Then we did the same comparison for the older age group; there was a difference with inferior retina being thinner than superior retina (P83–89 Sup: 57.9±5.5 μm; versus Inf: 51.6±3.4 μm; p = 0.006).

Histograms are shown of the photoreceptor laminar thickness (average across the vertical meridian) for the two age groups of mutant mice, expressed as a fraction of the earlier timepoint (Fig. 2B, lowest panel). Total retinal thickness was also measured in the scans from the two age groups and these results were consistent with those of the photoreceptor laminae; there was a significant difference between ages with older group thinner than the younger (P31–41: 197.4±13 μm; P83–89: 165.5±34.4 μm; p<0.001). Considering that these non-invasive imaging data represent the more central retina of the mouse eye, we also measured ONL thickness in histological sections from the peripheral retina (Fig. 2C). Histological sections of mutant mice at different ages also illustrate the differences in laminar architecture compared to WT. *Rd16;Nrl^−/−^* show pseudorosettes, limited IS/OS material and apparent retinal thinning over this time period (Fig. 2C). Two timepoints similar to those from the *in vivo* imaging studies were chosen and histograms of ONL thickness were plotted as a fraction of the earlier timepoint. In summary, there is a detectably thinner photoreceptor layer thickness at the older age using both methods and in different retinal regions (Fig. 2C).

### Pseudorosettes: Topographical Distribution and Age Effects

**Figure 3:**
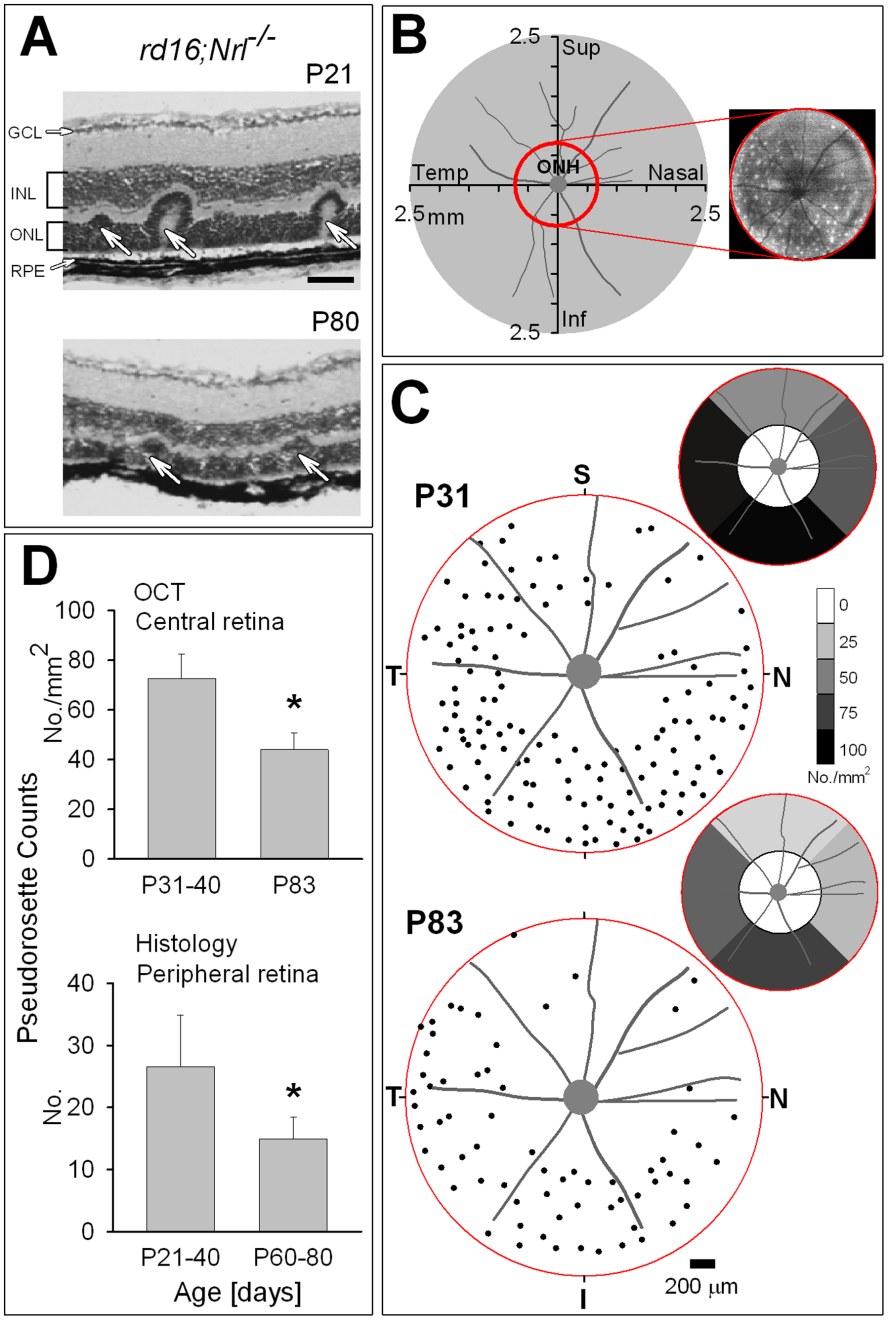
Spatial and temporal distribution of pseudorosettes in *rd16;Nrl^−/−^* retina. (A) Histological sections from peripheral retina of two *rd16;Nrl^−/−^* mice at different ages demonstrating the presence of pseudorosettes (arrows). Calibration = 50 μm. (B) Schematic drawing of the mouse retina indicating the coverage of the central OCT raster scans (red circle). ONH is centered in the drawing. Integrated *en face* image of the central region of a P41 *rd16;Nrl^−/−^* mouse showing how pseudorosettes appear as white dots (B, right panel, red circle). (C) Pseudorosette distribution within the central retinal region in a young (P31) and an older (P83) *rd16;Nrl^−/−^* eyes. Insets (up and right) show average pseudorosettes as density in different sectors of the central retinal region sampled (n = 8 eyes for both age groups). (D) Upper: Histograms comparing number of pseudorosettes in the central retina by OCT at two different ages (P31, n = 10 eyes; P83, n = 8 eyes). Lower: Pseudorosette counts from histological sections of peripheral retina of two different age groups (P21–40, n = 6 eyes; P60–80, n = 6 eyes). Both data sets in *rd16;Nrl^−/−^* mice indicate that the number of rosettes decreases with age (*represents *p*<0.001 and *p = *0.01 for the upper and lower graphs, respectively). Error bars, ± SD from the mean.

Histological sections from the peripheral retina in two *rd16;Nrl^−/−^* mice of different ages indicate the presence of pseudorosettes at both earlier and later timepoints (Fig. 3A). In the central retina (1.5 mm diameter) we quantified the number and location of pseudorosettes using *en face* imaging (Fig. 3B). Integration of backscatter intensity between boundaries enveloping all pseudorosettes in the raster images produced fundus images with white spots that represent the pseudorosettes [19–22} (Fig. 3B). Localization of individual pseudorosettes was then recorded with respect to the ONH. Pseudorosettes are detected in all quadrants of the retina in both younger (exemplified by a P31 eye) and older (exemplified by a P83 eye) *rd16;Nrl^−/−^* mice (Fig. 3C). In the older mutant mice, pseudorosettes appeared less dense but similar in pattern to the younger mutants. There was also an apparent regional variation with superior (dorsal) retina having a lesser number of these structures compared to the inferior (ventral) retina. A comparison of superior versus inferior pseudorosette counts in both age groups showed that the superior-inferior difference was significant (P31: n = 8 eyes, average number of pseudorosettes ± SD; Sup: 44±11 versus Inf: 73±12, p<0.001; P83: n = 8 eyes, Sup: 24±11 versus Inf: 44±13, p<0.001). These regional variations of the structures prompted us to examine the average density in different sectors within the central retina (Fig. 3C insets, n = 8 eyes for both age groups). Pseudorosette density was found to be higher in the inferior and temporal sectors than in the superior and nasal retina.

Two further comparisons were made – one by OCT in the central retina and the other by histology in the peripheral retina (Fig. 3D). The number of pseudorosettes in the central area sampled was compared statistically in the two age groups (Fig. 3D, upper histograms) and there was a significantly lower number in the older versus the younger mutants (p<0.001). Number of pseudorosettes were also counted in histological sections from the peripheral retina of the mutant mice and there was a significant difference between P21–40 and the P60–80 groups (p = 0.01) with, again, a reduced number of these structures at older ages (Fig. 3D, lower histograms).

### Expression of Phototransduction Proteins in *rd16;Nrl^−/−^* mice at Different Ages

**Figure 4:**
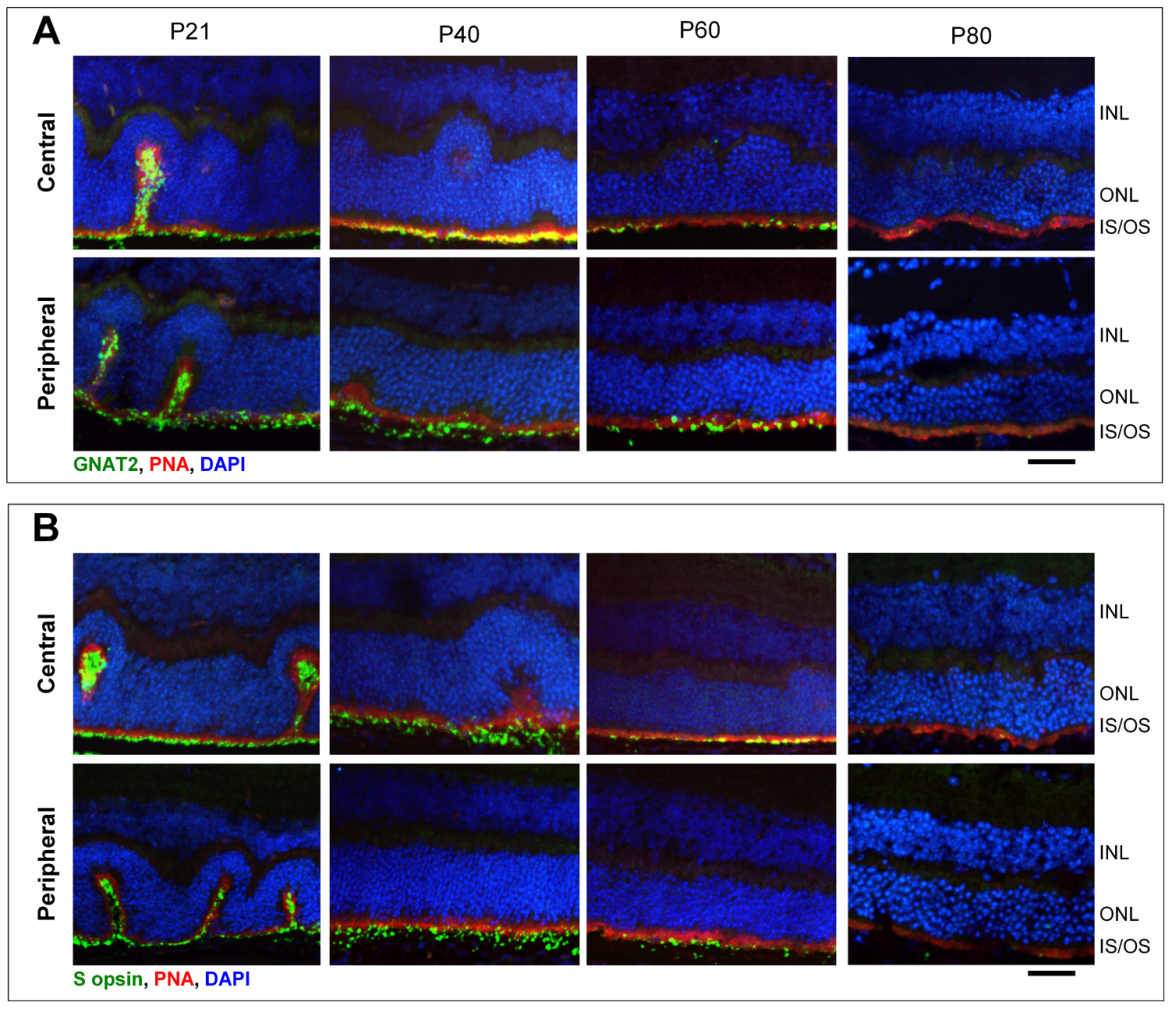
Expression of photoreceptor proteins is reduced over time in *rd16;Nrl^−/−^* mice. Representative cross sections (20X) from central and peripheral retina of P21, P40, P60 and P80 *rd16;Nrl^−/−^* mice were immunostained for the presence of cone transducin alpha (GNAT2) (A) or S- cone opsin (B) and PNA (A,B). Despite maintenance of cone outer segment sheaths (PNA), both GNAT2 and S-opsin expression are markedly reduced by P60 in both central and peripheral retina. INL- inner nuclear layer, ONL- outer nuclear layer, IS/OS- inner segments/outer segments. Calibration = 35 μm.

Previous studies have shown that loss of full length CEP290 expression leads to mislocalization and/or loss of photoreceptor proteins [7], [9], [23]. Here we evaluate whether this phenomenon is also observed on an *Nrl^−/−^* background. We show that expression of cone phototransduction proteins including the alpha subunit of cone transducin (GNAT2) and S-cone opsin decrease over time in the *rd16;Nrl^−/−^* mice. Loss of expression was evident in both central and peripheral retina (Fig. 4A,B). Regardless of age, both GNAT2 and S-opsin localized to remnant IS/OS of *rd16;Nrl^−/−^* photoreceptors. The most pronounced loss of both proteins appeared to occur between P40–P60, with very little of each detected via immunohistochemistry at P80. Despite loss of phototransduction proteins, cone outer segment sheaths, as detected by conjugation with peanut agglutinin (PNA), remained intact at all ages studied (Fig. 4A,B). This suggests that loss of phototransduction proteins in *rd16;Nrl^−/−^* is the result of their downregulation and/or increased turnover rather than loss of outer segments or photoreceptor degeneration.

### Cone Function in *rd16;Nrl^−/−^* mice and Relationship to Structure

**Figure 5:**
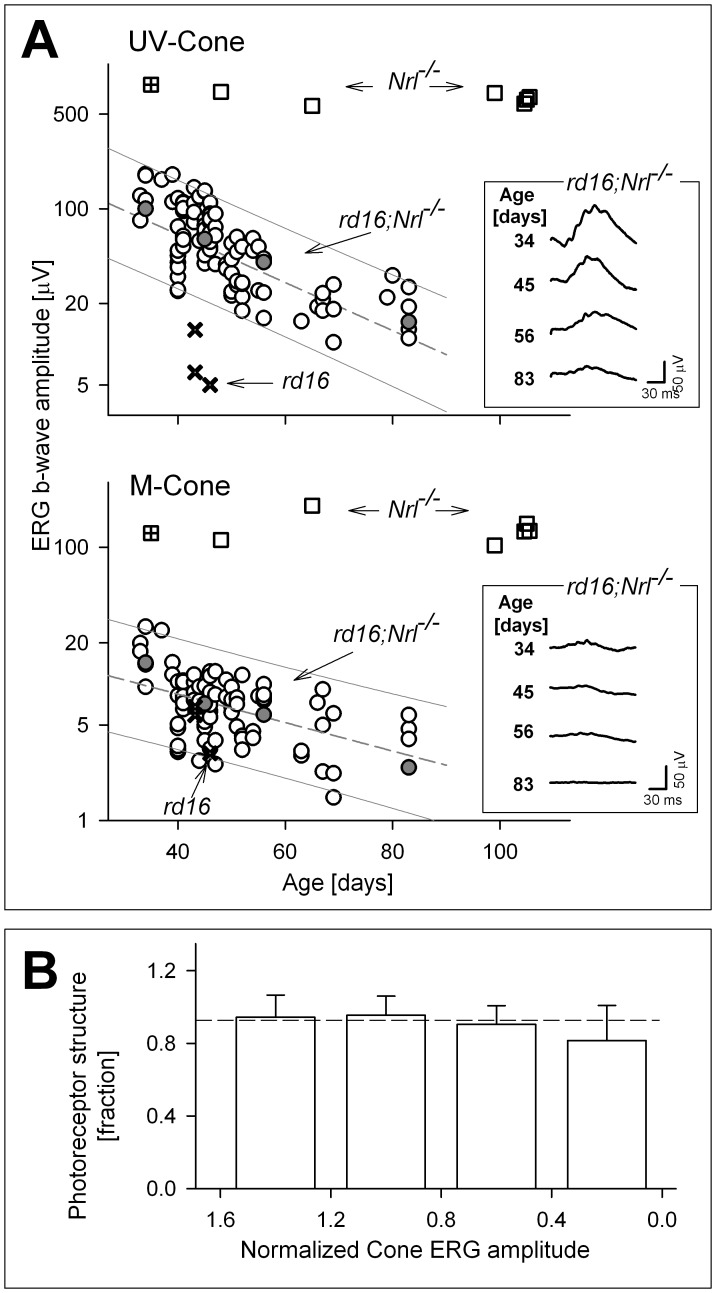
Structure and function in the *rd16;Nrl^−/−^* mouse retina. (A) ERG b-wave amplitudes of responses to UV- and M-cone stimuli as a function of age in *rd16;Nrl^−/−^* mice from P34 to P83 (n = 95) with comparisons to data from previously recorded signals in *Nrl^−/−^* (squares [11]; square with cross [19]), and *rd16* (crosses [7]) mice. Upper: Cone b-wave responses to ultraviolet (UV, 360 nm peak) stimuli in the *rd16;Nrl^−/−^* mice are severely reduced compared with those of *Nrl^−/−^* mice at comparable ages. Amplitudes in *rd16* mice are low compared to the other mice. It is also notable that ERGs of the *Nrl^−/−^* mice remain relatively stable throughout this age range, while ERGs of the *rd16; Nrl^−/−^* and *rd16* mice decline in amplitude with age. Lower: Responses to green (510 nm) stimuli are substantially lower in amplitude than those from UV-cone stimuli. Again, *Nrl^−/−^* mice have the largest amplitudes and do not decline with increasing age within this time period. The *rd16;Nrl^−/−^* waveforms are lower in amplitude and there is a reduction with age. Only limited data were available for *rd16* mice and these fell within the range of *rd16;Nrl^−/−^* amplitudes. Waveforms for representative *rd16;Nrl^−/−^* mice at various ages (grey-filled circles) are illustrated in the panels at right. Grey lines: linear regression fit to log-converted data (dashes) and 95% prediction intervals (solid). Squares with cross at earliest age in graphs: *Nrl^−/−^* data from Mears et al., 2001 (B) Photoreceptor structure (ONL+) as a function of the combined UV- and M-cone ERG b-wave amplitudes. ONL+ remains similar to the value at P31 (youngest age *rd16;Nrl^−/−^* we studied) across various degrees of ERG amplitude reduction. Horizontal dashed line is the reference level for the lower limit of retinal structure thickness at P31 (−2SD from the mean at this age); photoreceptor structure above this lower limit indicates no difference compared to the data of P31 (error bars, +2SD from mean).

ERG responses elicited with UV- and M-cone stimuli in *rd16;Nl^−/−^* mice over the age range from ∼30 to ∼90 days of age are illustrated and b-wave amplitudes are plotted (Fig. 5A). Responses from a limited number of *Nrl^−/−^* [11], [19] and *rd16* [7] mice are shown for comparison. UV-cone responses in the *Nrl^−/−^* mice are ∼1 log-unit higher in amplitude than those in *rd16;Nrl^−/−^* at the younger ages depicted. The *Nrl^−/−^* responses show no change over the time period studied. In contrast, the *rd16* mice have responses that are almost reaching noise levels by day 44, and are ∼1 log unit lower in amplitude than the *rd16;Nrl^−/−^* at that age. Between these two extremes are the data from the *rd16;Nrl^−/−^* mice, which show a measurable decline from an average of 139 μV (range, 103–210 μV) at 31 days to 22 μV (range, 13–36 μV) by age 83 days. The natural history of function loss is consistent with an underlying exponential decay which shows linear reduction of amplitudes on log-linear plots (Fig. 5A).

M-cone ERGs in the *rd16;Nrl^−/−^* mice are substantially lower in amplitude than the UV-cone ERGs. The *Nrl^−/−^* mice show responses about 1 log unit higher in amplitude than those of the double mutants at the earlier ages and there is no reduction in amplitude over the ages sampled. Limited data from *rd16* mice were recordable before 44 days of age and within the range of response amplitudes of the double mutants. In *rd16;Nrl^−/−^* mice there is a measurable decline of M-cone ERG amplitude with age (Fig. 5A).

The structure-function relationship we plotted for human *CEP290*-LCA (Fig. 1B) can now be compared to a structure-function relationship in the *rd16;Nrl^−/−^* mouse model (Fig. 5B). Despite very different types of data and measurements, we tried to plot the human results for structure (by OCT) and function (by visual acuity) and the mouse data for structure (OCT) and function (by ERG amplitudes) in a way that allowed some comparison, even if by simple observation. Photoreceptor layer (ONL+) thickness and cone ERG amplitudes (using the sum of UV- and M-cone ERG amplitudes) were normalized to the earliest timepoint studied (P31). Cone ERGs in the double mutant, compared with the all-cone *Nrl^−/−^* mice without the *Cep290* mutation, are quite reduced in amplitude and, like the visual acuities of *CEP290*-LCA, show a range of abnormalities that only worsens over the age period studied (Fig. 5A). Cone photoreceptor structure shows change but it is relatively limited, only becoming evident at the lowest ERG amplitudes (Fig. 5B).

## Discussion

### Developing a Strategy for Proof-of-concept Studies

What did we learn from the studies of the *rd16;Nrl^−/−^* model of *CEP290*-LCA that would guide strategy for proof-of-concept research using, for example, gene augmentation therapy by subretinal delivery? The relatively persistent cone photoreceptor cell layer thickness over the first three months of life and the measurable ERG responses during these ages provides an opportunity for intervention. What are the factors that constrain the timing of intervention and of reassessment to determine efficacy or toxicity? The slow loss of ONL thickness during this interval would not be limiting. The declining ERG amplitudes, the loss of phototransduction proteins, and the onset time for transgene expression of the viral vector, however, would dictate the timing of intervention.

The ERG response to assay, given the major difference between UV- and M-cone amplitudes, is the UV-cone b-wave which declined at a rate of about 4% per day over the period from 1 to 3 months of age. The basis for the loss of cone amplitude was not able to be determined in this study, but likely contributors could be changes in the length of the already diminutive outer segments and diminishing phototransduction proteins therein. Our data suggest that a uniocular subretinal injection of vector-gene occur at age P35–40, when UV-cone ERG responses are still substantial: mean 84 μV (1.9±0.4 log μV, 95% prediction interval). Four weeks thereafter, at P65, there would be functional assessment with bilateral ERGs. At this age, responses in the untreated eye would be expected to be lower but clearly distinguishable from noise levels: mean 25 μV (1.4±0.4 log μV). Interocular asymmetries (±0.3 log) [24] could then be used to assess treatment efficacy (>50 μV) or toxicity (<12 μV). Such a strategy of interocular difference assessment by ERG has been used in prior murine studies of therapeutic efficacy [24]. Of course, an earlier age for initiation of therapy is also possible but we have no data to support that recommendation.

What are other uncertainties in this theoretical experiment? Subretinal injections and resulting retinal detachments are well-known to have negative effects on photoreceptor outer segments as a result of trauma [25], [26]. It has not been established from earlier work how fragile the shortened outer segments are in either *Nrl^−/−^* mice or *rd16;Nrl^−/−^* double mutants. *Nrl^−/−^* mice have been used to engineer cone-only mouse mutants with other genetic abnormalities [27]–[29]. It is promising that GFP expression has been obtained in *Nrl^−/−^* mice with subretinal AAV [30], and preliminary results suggest efficacy after subretinal gene therapy in an *Nrl^−/−^;Cnga3^−/−^* double mutant [31].

Another issue to be addressed is the dysplastic phenotype, i.e. the presence of pseudorosettes, that we quantified in this study. From the present results, the superior retina showed less numbers of pseudorosettes at the early and later ages sampled. The finding of less degeneration in the superior retina would be consistent with results of previous studies suggesting a relationship of the dysplastic phenotype and eventual degeneration in mouse mutants including *Nrl^−/−^mice* [12], [19], [20], [22]. If technically feasible to place subretinal injections mainly in the superior retina, it should avoid the greater complexity of the inferior retina where we showed a higher density of pseudorosettes. Targeting treatment to specific retinal regions in humans undergoing subretinal gene therapy has been valuable in the *RPE65*-LCA trials [4]; in this *rd16;Nrl^−/−^* mouse mutant, it would seem worthy to attempt, if surgically possible.

### Viral Vector Considerations for Eventual Delivery of *Cep290* to Cone Photoreceptors

Which vector platform will be useful for proof-of-concept gene replacement studies in *rd16;Nrl^−/−^* mice? Choice must be dictated at least by the ability of vector to transduce the target cell (photoreceptors) and mediate persistent transgene expression. If delivery of full length Cep290 is necessary (cDNA = ∼7.4 kb), vector must also accommodate a relatively large genetic payload. Lentivirus can accommodate full length Cep290 cDNA. Equine infections anemia virus (EIAV)-based lentivirus, used in concert with various photoreceptor-specific promoters, showed modest expression of reporter gene in photoreceptors of mice subretinally injected at postnatal day 5 (P5), a timepoint prior to photoreceptor differentiation [32]. EIAV containing photoreceptor-specific *ABCA4* (another relatively large cDNA) restored the disease phenotype of *Abca4^−/−^* mice following subretinal injection at P4–P5 [33]. Transduction of postmitotic photoreceptors has been more challenging in rodents [34]–[39] but not in primates [40].

Adeno-associated virus (AAV) has proven useful for driving persistent therapeutic transgene expression in postmitotic photoreceptors [41]–[44] and has demonstrated safety and efficacy in proof-of-concept studies [45] as well as clinical trials [46]–[49]. The Cep290 cDNA exceeds the packaging capacity of conventional AAV (∼5 kb). Recently, however, it was reported that AAV is capable of delivering large cDNAs. Several groups demonstrated that large cDNAs are randomly truncated during packaging [50]–[53]. Upon co-infection, fragmented genomes most likely undergo homologous recombination leading ultimately to expression of full length protein. Fragmented AAV (“fAAV”) has been used successfully to correct the retinal phenotypes of multiple mouse models of inherited retinal disease [54]–[56]. Because the heterogeneous nature of genetic payloads in “fAAV” vectors may limit their therapeutic application, various dual AAV vector platforms in which single, defined DNA species are encapsidated (vector 1 containing the 5′ portion of the cDNA and vector 2 containing the 3′ portion of the cDNA) have been developed. Such platforms have proven useful for delivery of a variety of large cDNAs [57]–[65].

What if the entire CEP290 protein (2479 amino acids) was not required for treatment? Recently, a zebrafish model of *CEP290*-LCA was created using morpholinos to generate an altered *cep290* splice product that creates a premature stop codon at amino acid 988, modeling the most common *CEP290*-LCA mutation [66]. Mutants displayed reduced visual function and delays in melanosome transport. Embryonic delivery of the N’- terminal portion alone (the first 1059 amino acids) was sufficient to restore the vision defect in this model [66]. A C’ terminal fragment (a.a. 1765–2479) proved useful only for restoring melanosome transport. Unlike the N’ terminal mutation in zebrafish, the *rd16* mouse carries a mutation close to the C’ terminus of Cep290 (in frame deletion of amino acids 1599–1897) [9], suggesting that this is the region of the protein required for vision. Reasons for this discrepancy have yet to be elucidated. It is possible that the *rd16* deletion mutation alters Cep290 folding, preventing the N’ terminus from performing an essential function.

It is known that the C’ and N’ termini of CEP290 can interact with themselves and one another [67]. A recent report shows that CEP290 activity is auto-inhibited via interactions between its N’ and C’ termini [68]. Full length CEP290 exhibited attenuated activity in hTERT-RPE1 cells whereas overexpression of N’ or C’ terminal portions alone resulted in aberrant primary cilium formation. Taken together, these studies suggest that the N’ and C’ termini physically interact to inhibit CEP290 function. Overexpression of N’ or C’ terminus alone in this cell line (which expresses endogenous full length protein), likely led to saturation of full length CEP290 regulatory domains and a resulting increase in protein function. It was suggested that truncation mutants of CEP290 that lack inhibitory domains but maintain other functional regions of the protein may prove useful (and perhaps even fit inside a conventional AAV vector) for the treatment of this disease. Future proof-of-concept studies with lentivirus, “fAAV”, dual AAV vector platforms carrying full length CEP290, and standard AAV carrying CEP290 truncation mutants are certainly warranted. The current work defines a specific strategy to evaluate the ability of such vectors to restore the retinal phenotype of the *rd16;Nrl^−/−^* mouse.
